# Sustainable Upcycling of Swine Wastewater Sludge: Using Thermal and Citrate Pretreatment to Enhance Volatile Fatty Acid Production

**DOI:** 10.3390/ani16091403

**Published:** 2026-05-03

**Authors:** Wei-Chen Chen, Jung-Jeng Su

**Affiliations:** 1 Department of Animal Science and Technology, National Taiwan University, Taipei 106319, Taiwan; d10626010@ntu.edu.tw; 2 Bioenergy Research Center, College of Bio-resources and Agriculture, National Taiwan University, Taipei 10617, Taiwan; 3 Agricultural Net-Zero Carbon Technology and Management Innovation Research Center, National Taiwan University, Taipei 106319, Taiwan

**Keywords:** swine wastewater sludge, volatile fatty acids, thermal–citrate pretreatment, divalent cation bridging, resource recovery, circular bioeconomy

## Abstract

The sustainable management of intensive swine farming is currently bottlenecked by the difficult valorization of metal-rich wastewater sludge. The structural rigidity of this sludge, stabilized by divalent cation bridging, severely limits its anaerobic digestion and overall resource recovery. To optimize the manure management chain, this study comprehensively evaluated various physical and chemical pretreatments to identify the most effective disintegration strategy for enhanced volatile fatty acid (VFA) production. Among the tested conditions, the coupling of thermal hydrolysis with citrate chelation (T/SC) was the most effective, achieving the highest disintegration degree (12.37%) and biopolymer solubilization. Mechanism analysis revealed that, unlike traditional alkaline treatments, which are limited by the severe reprecipitation of magnesium and phosphate, citrate effectively sequestered bridging cations (Ca^2+^ and Mg^2+^) via ligand exchange. This synergistic disintegration accelerated the fermentation kinetics, enhancing the total VFA yield 2-fold (1293 mg/L) compared to the control group while maintaining a high-value, butyrate-dominant product profile. These findings demonstrate that targeting ionic bridges via ligand-promoted dissolution provides a highly practical and sustainable strategy to maximize resource recovery and nutrient cycling from metal-laden livestock wastes.

## Simple Summary

Pig farm wastewater sludge is difficult to recycle because metals lock the waste particles tightly together. After exploring various traditional treatments, this study discovered that combining heat with citrate effectively breaks these strong metal bonds. This specific combination successfully unlocked the trapped organic materials, allowing bacteria to achieve the highest production of valuable chemicals called volatile fatty acids. These chemicals can serve as sustainable raw materials for manufacturing eco-friendly plastics. Ultimately, this targeted approach offers a practical solution to transforming tough agricultural waste into valuable resources, significantly reducing environmental pollution and promoting a sustainable farming economy.

## 1. Introduction

Anaerobic fermentation of organic wastes for volatile fatty acid (VFA) production is a promising valorization route [1,2]. However, the overall efficiency is severely restricted by the slow hydrolysis of solid organic matter, which is widely recognized as the rate-limiting step [3,4]. This limitation is particularly pronounced in swine wastewater sludge (SMS). SMS, collected from an on-site livestock wastewater treatment facility, consists of a complex mixture of anaerobic digested sludge and waste activated sludge generated from a typical three-stage treatment process. The rigid cell walls and compact floc structures of these microbial cells act as severe physical barriers to enzymatic attack, resulting in low biodegradability without effective pretreatment [5,6]. Furthermore, unlike typical municipal sludge, SMS is characterized by an exceptionally high content of divalent cations (e.g., Ca^2+^ and Mg^2+^) and heavy metals (e.g., Cu^2+^ and Zn^2+^) derived from intensive feed additives [7]. These metal ions complex tightly with extracellular polymeric substances (EPS), forming a rigid metal-bridging network (Divalent Cation Bridging, DCB) that contributes significantly to the structural integrity of the mixed sludge, making it highly recalcitrant to biodegradation [8,9].

To enhance sludge disintegration, alkaline pretreatment (pH > 10) is widely applied. However, for phosphorus-rich and metal-laden substrates like SMS, alkaline treatment is often counterproductive. Then literature indicates that elevated pH levels trigger the rapid reaction of released orthophosphate with co-existing calcium and magnesium ions, resulting in the formation of insoluble precipitates such as calcium phosphate and struvite [10,11]. This reprecipitation phenomenon not only locks valuable phosphorus within the solid residue, hindering downstream recovery [12], but also causes scaling issues. Moreover, physical treatments alone (e.g., thermal hydrolysis) often require excessive energy input to disrupt the strong electrostatic forces governed by divalent cation bridging. Therefore, a targeted strategy that can selectively disrupt these ionic bridges without causing precipitation is urgently needed.

To address these specific limitations, this study proposes a coupled chemical–physical pretreatment strategy utilizing sodium citrate (SC). Sodium citrate was selected specifically as a ligand exchange chelator to sequester bridging cations (Ca^2+^ and Mg^2+^) from the EPS network into soluble complexes [13], thereby preventing mineral precipitation. While SC is expected to fundamentally unlock the ionic barrier, auxiliary physical energy is required to accelerate the hydrolysis kinetics and maximize structural disintegration. However, it remains unclear whether thermal (T) or ultrasonic (U) energy provides a superior synergistic effect when coupled with citrate. Therefore, the main aim of this work is to comprehensively evaluate and compare these coupled strategies (i.e., T/SC versus U/SC) against conventional methods to identify the optimal pathway for overcoming the specific ionic barrier of SMS. We hypothesize that citrate will act as a powerful ligand to induce profound structural disruption without reprecipitation, and that its combination with the appropriate physical energy will dictate the ultimate pretreatment efficacy. The subsequent effects of this targeted disruption on the metabolic pathways and the enhancement of high-value VFA production were systematically investigated.

## 2. Materials and Methods

### 2.1. Swine Wastewater Sludge Sampling and Characterization

The sludge used in this study was collected from the gravity-thickened sludge tank at the swine wastewater treatment facility in Taipei, Taiwan. The farm employs a modified three-stage livestock wastewater treatment process. Initially, primary solid–liquid separation is applied to remove coarse feces. The liquid fraction then undergoes anaerobic digestion for bulk organic reduction and biogas production. For the final aerobic polishing stage, a sequencing batch reactor (SBR) is utilized instead of the conventional continuous-flow aeration tank and secondary clarifier. The SBR fundamentally maintains the aerobic activated sludge process but effectively integrates biological degradation and sludge settling within a single reactor operating in sequential time cycles. The sludge used in this study was collected from the gravity-thickened sludge tank, which gathers the excess biosludge generated from both the anaerobic digester and the SBR. Upon collection, the sludge was screened through a 2.0 mm sieve to remove coarse debris and stored at 4 °C before use. The main physicochemical characteristics of the raw sludge are summarized in Table 1.

### 2.2. Pretreatment Methods

Eight different pretreatment strategies were evaluated, including chemical (sodium citrate [SC], alkaline [A]), physical (thermal [T], ultrasonic [U]), and combined physical-chemical methods (T/SC, T/A, U/SC, and U/A). The experiments were conducted in 150 mL laboratory bottles (Duran Schott, Mainz, Germany) with a working volume of 60 mL. Detailed operating conditions are listed in Table 2.

For physical pretreatments, T was performed by heating the sludge at 121 °C for 30 min [14]. U was conducted using an ultrasonic processor at 40 kHz (300 W) for 1 h [15]. For chemical pretreatment, A was achieved by adjusting the sludge pH to 12.0 with 4 M NaOH, followed by agitation at 300 rpm for 1 h [16]; the pH was then neutralized to 7.0 with 4 M HCl. For SC, sodium citrate (Sigma-Aldrich, St. Louis, MO, USA) was dosed at 0.3 g/g-TS, and the mixture was stirred at 150 rpm for 1 h at room temperature (25 ± 2 °C) [13].

For combined pretreatments, although the abbreviation places the physical method first (e.g., T/SC, U/SC) to denote the primary energy input, the chemical agent was consistently dosed and mixed before the physical treatment. In T/SC, sodium citrate (0.3 g/g-TS) was added and stirred for 30 min before thermal treatment (121 °C, 30 min). In T/A, the sludge pH was adjusted to 12.0 and stirred for 30 min, followed by thermal treatment; the pH was then neutralized to 7.0 with HCl. In U/SC, the sludge mixed with SC was subjected to ultrasonic treatment for 1 h. Similarly, in U/A, the pH-adjusted sludge (pH 12) was sonicated for 1 h. Following pretreatment, pH, electrical conductivity (EC), soluble protein (PN), soluble polysaccharides (PS), total chemical oxygen demand (TCOD), soluble chemical oxygen demand (SCOD), and ionic species were analyzed.

### 2.3. Batch Fermentation Experiment

Anaerobic sludge as the inoculum was obtained from the anaerobic digester of the wastewater treatment plant. To selectively enrich spore-forming hydrolytic and acidogenic bacteria while inactivating methanogens, the fresh anaerobic sludge was thermally pretreated at 100 °C for 30 min [17]. The heat-treated sludge was washed three times with distilled water by centrifugation at 8000 rpm for 5 min to remove residual metabolites. The pellet was then resuspended in distilled water to its original volume.

**Figure A1:**
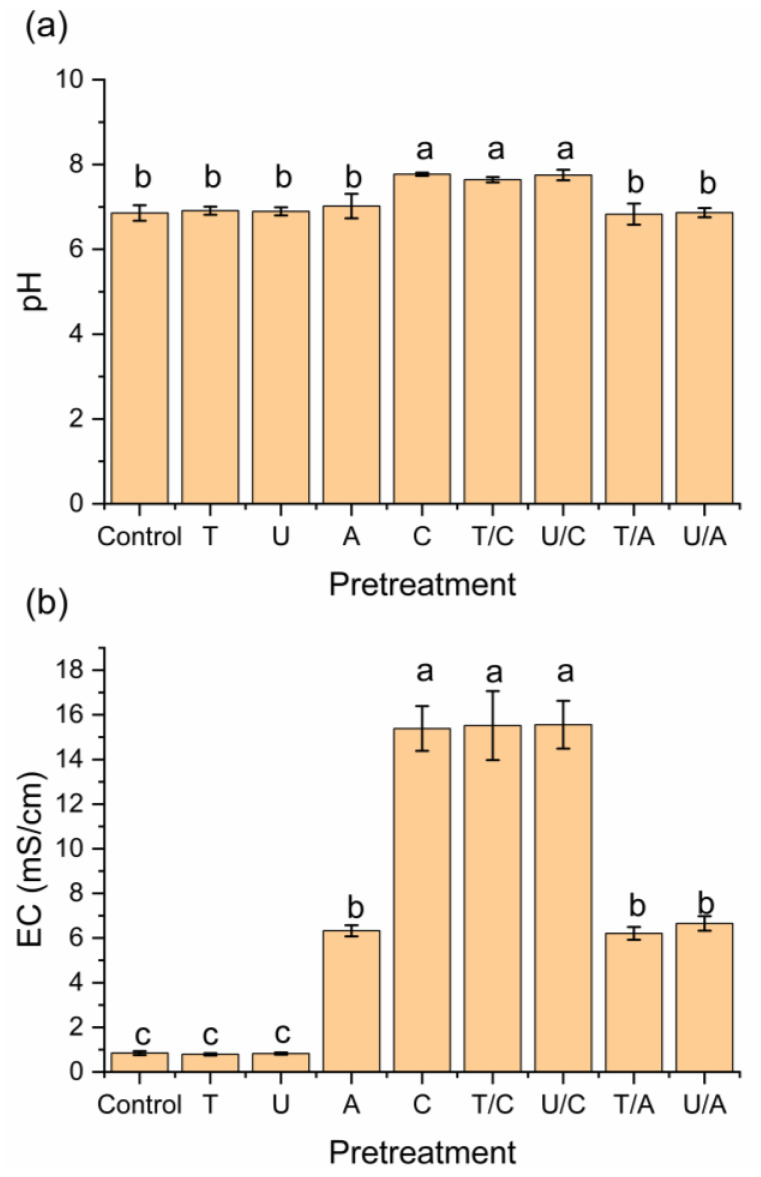
Variations in (**a**) pH value and (**b**) electrical conductivity (EC) of livestock sludge after different pretreatments. Mean ± SD (*n* = 6). Different letters denote significant differences (*p* < 0.05, Tukey’s HSD). T = thermal; U = ultrasonic; A = alkaline; SC = sodium citrate.

Anaerobic batch fermentation was carried out in 250 mL serum bottles. Each reactor contained 150 mL of pretreated sludge (or untreated sludge for the control) and 23.2 mL of the prepared inoculum (resulting in a substrate-to-inoculum volume ratio of approximately 6.5:1). The pH profiles of the initial fermentation process are provided in Appendix Figure A1. To ensure anaerobic conditions, the headspace was flushed with nitrogen gas for 5 min, and a 1 L gas bag (SKC Inc., Eighty Four, PA, USA) filled with 100 mL of nitrogen was connected to each bottle to balance internal pressure. The reactors were incubated at 37 °C in a shaker at 100 rpm for 10 days. Samples were collected daily to monitor VFA production.

### 2.4. Analysis Methods

Total solids (TS), volatile solids (VS), TCOD, and SCOD were determined according to Standard Methods [18]. The degree of disintegration (*DD*_COD_) was calculated using Equation (1) [19]:

(1) $${DD}_{COD}\left(\%\right)=\frac{{SCOD}_{treated}-{SCOD}_{raw}}{{TCOD}_{raw}-{SCOD}_{raw}}\times 100$$

To correct for the interference of sodium citrate (a soluble organic compound) on COD measurements, the COD of the sodium citrate solution was experimentally determined and subtracted from the measured TCOD and SCOD values in SC-based pretreatments. PN and PS were quantified using the modified Lowry method [20] and the phenol–sulfuric acid method [21], respectively, with bovine serum albumin and glucose as standards. Conversion factors of 1.50 g-COD/g-protein and 1.07 g-COD/g-carbohydrate were used to calculate their COD equivalents [22].

The pH and EC were measured with standard meters (PH200 and CON200, CLEAN Instruments Co. Ltd., New Taipei City, Taiwan). Ion concentrations (Cations: Na^+^, K^+^, Ca^2+^, NH_4_^+^, Mg^2+^, anions: Cl^−^, NO_2_^−^, NO_3_^−^, PO_4_^3−^, SO_4_^2−^) were analyzed via ion chromatography (883 Basic IC Plus, Metrohm, Herisau, Switzerland). VFA composition (C2–C5) was quantified using a gas chromatograph (7820A, Agilent, Santa Clara, CA, USA) equipped with a flame ionization detector (FID) and a Nukol capillary column (30 m × 0.25 mm × 0.25 µm, Supelco, Merck KGaA, Darmstadt, Germany). Nitrogen was used as the carrier gas. The oven temperature was programmed as follows: initiated at 80 °C, increased to 180 °C at 10 °C/min, and held for 5 min. Crotonic acid served as the internal standard [23].

### 2.5. Statistical Analysis

Statistical analyses and graphical representations were performed using Origin 2020b (OriginLab Corporation, Northampton, MA, USA). One-way analysis of variance (ANOVA) followed by Tukey’s honestly significant difference (HSD) post hoc test was used to compare means among treatment groups. Differences were considered statistically significant at *p* < 0.05. Additionally, a two-way ANOVA was conducted to evaluate the main and interactive effects of physical and chemical pretreatment factors on sludge solubilization and VFA production. Data are presented as mean ± standard deviation (SD).

## 3. Results and Discussion

### 3.1. Impact of Pretreatments on Sludge Solubilization and Disintegration

**Figure 1:**
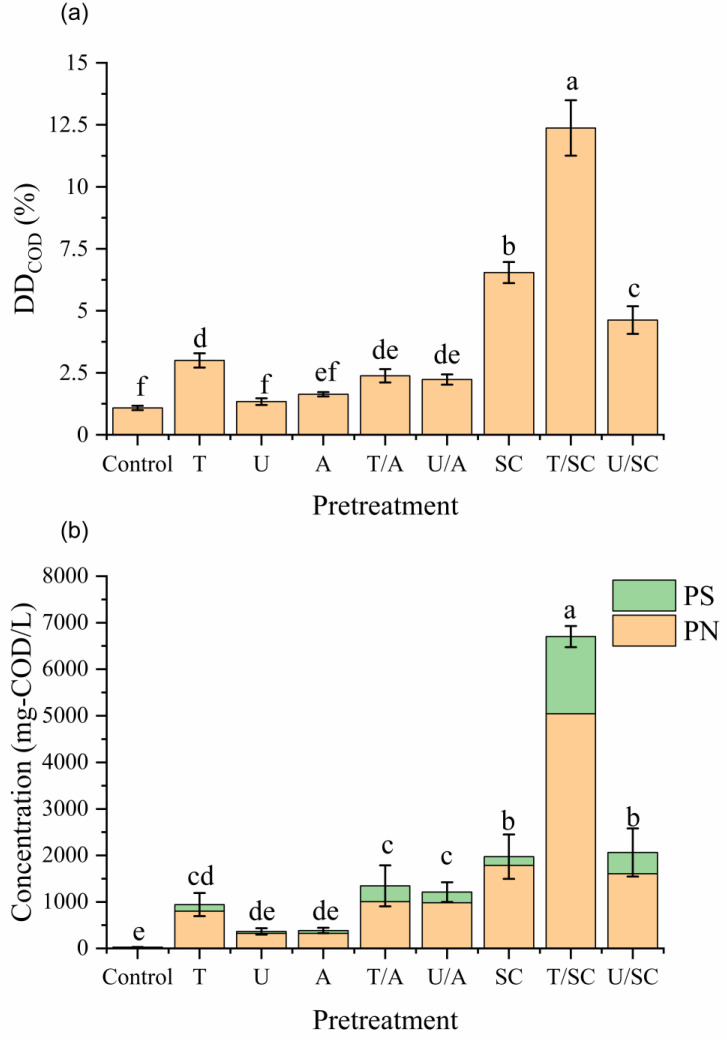
Effects of physical, alkaline, and citrate pretreatments on (**a**) disintegration degree (DD) and (**b**) soluble protein (PN) and polysaccharide (PS) concentrations. Values are mean ± SD (*n* = 6). Different letters denote significant differences (*p* < 0.05, Tukey’s HSD). T = thermal; U = ultrasonic; A = alkaline; SC = sodium citrate.

**Figure 2:**
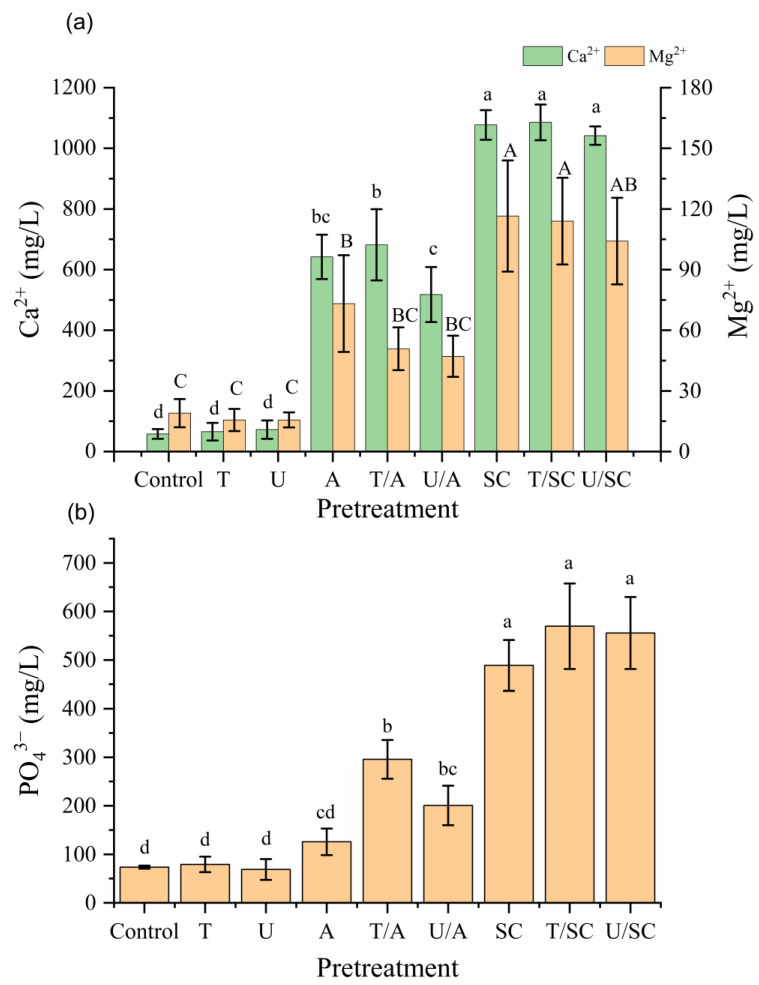
Effects of physical, alkaline, and citrate pretreatments on (**a**) Ca^2+^, Mg^2+^ removal and (**b**) PO_4_^3−^ from livestock sludge. Green and orange bars in (**a**) show Ca^2+^ (left axis) and Mg^2+^ (right axis). Values are mean ± SD (*n* = 6). Different lowercase letters (for Ca^2+^ and PO_4_^3−^) and uppercase letters (for Mg^2+^) denote significant differences (*p* < 0.05, Tukey’s HSD). T = thermal; U = ultrasonic; A = alkaline; SC = sodium citrate.

The efficacy of different pretreatment strategies was evaluated using disintegration degree (*DD*_COD_), which normalizes the solubilized organic matter to the total organic load (Figure 1a). Since TCOD remained relatively stable across all groups (55,736 ± 2690 mg/L), the *DD*_COD_ values directly reflect the solubilization efficiency. In the control group, the *DD*_COD_ was approximately 1.08 ± 0.09% (equivalent to an SCOD of 580 ± 44 mg/L). Statistical analysis revealed distinct performance tiers among the treatments. Surprisingly, neither Ultrasonic (U) nor Alkaline (A) pretreatment alone resulted in a significant increase in solubilization compared to the control (*p* > 0.05). This indicates that the rigid floc structure of this gravity-thickened swine sludge is resistant to moderate mechanical cavitation and insensitive to pH adjustment alone. Unlike municipal sludge, where physical disruption or EPS removal strategies have been reported to enhance solubilization effectively [24], the relative ineffectiveness of the U/A and T/A strategies in this study, coupled with the massive release of Ca^2+^ and Mg^2+^ during SC treatment, strongly implies that divalent cation bridging acts as a major structural barrier in this metal-rich swine sludge. Additionally, the sludge’s inherent high buffering capacity likely neutralized the chemical impact of alkaline addition. In contrast, Thermal (T) and Sodium Citrate (SC) pretreatments significantly enhanced disintegration (*p* < 0.05) [19,25,26]. The SC-based strategies demonstrated the most dominant performance, securing the top three positions in terms of solubilization efficiency (T/SC > SC > U/SC). The highest disintegration was achieved in the T/SC group, with a *DD*_COD_ of 12.37 ± 1.12% (SCOD: 6906 ± 449 mg/L). It is noteworthy that SC alone (the second-highest) achieved a higher *DD*_COD_ (6.54 ± 0.43%) than the combined T/A and U/A groups. This suggests that the specific chelation of bridging metals by citrate is a primary driver of floc disintegration in this metal-rich sludge. The substantial release of Ca^2+^ and Mg^2+^ (Figure 2) aligns with the cation exchange mechanism, wherein citrate likely displaces divalent structural ions with monovalent Na^+^. Based on similar observations in waste activated sludge [14], this ion exchange is hypothesized to induce the swelling and subsequent disruption of the EPS matrix [8,9].

To further understand the nature of the solubilized organics, the concentrations of PN and PS, the major components of EPS and intracellular fluids, were analyzed (Figure 1b) [27]. The release patterns of PN and PS largely mirrored the SCOD trends, with distinct stepwise increases across treatment tiers. In the Control, U, and A groups, the concentrations of PN and PS were relatively low, with no statistically significant differences observed among them (*p* > 0.05). This reaffirms that moderate ultrasonic cavitation or pH adjustment alone was insufficient to effectively disrupt the rigid EPS matrix of the gravity-thickened sludge to release biopolymers. A significant improvement was observed in the T group compared to the control (*p* < 0.05). However, the enhancement provided by thermal treatment appeared to reach a plateau; although the combined alkaline groups (T/A, U/A) showed significantly higher release than their single counterparts (A, U), they did not statistically surpass the single T group (*p* > 0.05). This places T, T/A, and U/A in the same intermediate efficacy tier (*DD*_COD_ ranges: 1.9–3.4%), suggesting that adding alkali provides negligible benefit over thermal hydrolysis for polymer release. This implies that the fraction of EPS accessible to thermal solubilization is limited, and the remaining recalcitrant structure, stabilized by metal bridges, cannot be accessed simply by increasing pH [8,9]. Furthermore, the sludge’s inherent high buffering capacity rapidly neutralized the chemical impact of the initial alkaline addition, as evidenced by the continuous pH monitoring (Figure A1a).

In contrast, the introduction of sodium citrate broke this ceiling. The SC-based groups (SC, U/SC, and T/SC) significantly outperformed the intermediate tier, releasing substantially higher amounts of PN and PS. The highest concentrations were observed in the T/SC group (PN: 5040 ± 244 mg-COD/L; PS: 1661 ± 130 mg-COD/L), which was statistically superior (*p* < 0.05). Crucially, the elevated concentrations of soluble PN and PS in the T/SC group represent a significant increase in readily biodegradable substrates, which is expected to facilitate downstream acidogenesis and VFA production [1,2].

#### 3.1.1. Role of Divalent Cation Chelation

To elucidate the mechanisms of disintegration and verify the critical role of divalent cations in maintaining sludge floc integrity, the release profiles of Ca^2+^ and Mg^2+^ were analyzed (Figure 2a) [8,9]. The release patterns of these two metals showed distinct trends, revealing fundamental differences in the ability of pretreatment strategies to disrupt ionic bridging. Regarding Ca^2+^ release, the treatments were statistically stratified into three distinct tiers. The Control, T, and U groups constituted the lowest tier with no significant differences among them (*p* > 0.05), indicating that physical shear or thermal energy alone was insufficient to disrupt the strong calcium-mediated linkages within the floc matrix. The alkaline-based groups (A, T/A, and U/A) formed an intermediate tier, showing moderate solubilization. However, the most significant release was observed in the citrate-based groups (SC, T/SC, and U/SC), which statistically outperformed both the physical and alkaline tiers (*p* < 0.05). Specifically, the soluble Ca^2+^ concentration in the SC groups surged to approximately 1077–1085 mg/L, representing an 18-fold increase compared to the Control (58.0 ± 16.0 mg/L). Notably, no significant differences were found among the T/SC, SC, and U/SC groups, suggesting that citrate was the sole determinant of effective calcium sequestration, regardless of the auxiliary physical energy. In contrast, the release pattern of Mg^2+^ exposed the specific limitations of alkaline treatment. Unlike Ca^2+^, the combined alkaline groups (T/A, U/A) failed to demonstrate significant improvement over the control, dropping to the same statistical level as the physical treatment groups (Control, U, and T) (*p* > 0.05). This inefficiency is likely due to rapid reprecipitation of solubilized magnesium as insoluble magnesium hydroxide or struvite at high pH [10,11]. Conversely, the citrate-based strategies maintained their dominance, securing the highest ranking for Mg^2+^ release (104–116 mg/L), with no significant intra-group differences. This confirms that citrate possesses a powerful, non-selective chelating ability that effectively overcomes both calcium and magnesium bridges [13,28]. Collectively, the relative ineffectiveness of the U/A and T/A strategies in releasing biopolymers (Figure 1b), coupled with the massive release of Ca^2+^ and Mg^2+^ during SC treatment (Figure 2a), strongly implies that divalent cation bridging acts as a major structural barrier in this metal-rich swine sludge [8,9]. The inability of physical treatments to mobilize cations confirms that floc disintegration requires chemical bond breakage rather than mechanical disruption. Furthermore, the superior performance of citrate over alkali is driven by ligand exchange, which actively sequesters metal ions from the EPS matrix and inorganic precipitates, preventing the reprecipitation issues observed in alkaline treatments [13,28].

#### 3.1.2. Release of Intracellular Markers and Inorganic Precipitates (PO_4_^3−^) Release

To investigate the extent of cell lysis and the dissolution of inorganic precipitates, orthophosphate (PO_4_^3−^) release was analyzed (Figure 2b). The results showed a clear grouping pattern driven primarily by the chemical agent used rather than the physical pretreatment. Statistical analysis revealed that T, U, and A treatments alone yielded negligible improvements in phosphate release compared to the Control group (*p* > 0.05). The inability of thermal or alkaline treatment to significantly elevate soluble phosphate levels suggests that these methods are limited by reprecipitation kinetics. On their own, they cannot effectively solubilize inorganic phosphate precipitates (e.g., calcium phosphate or struvite) prevalent in the sludge, nor can they maintain the intracellular phosphorus released from cell lysis in the soluble phase, likely due to rapid scavenging by free metal ions [10,11]. A slight numerical increase was observed in the combined alkaline groups (T/A, U/A) (reaching 200–295 mg/L), which were statistically comparable to the A group but significantly lower than the citrate-based groups. In contrast, all citrate-based groups (T/SC, U/SC, SC) achieved the highest phosphate release (489–570 mg/L), forming the statistically superior tier. However, a critical finding is that there were no significant differences among the T/SC, U/SC, and SC groups (*p* > 0.05). This indicates that sodium citrate is the sole governing factor for phosphate solubilization in this system. The addition of thermal or ultrasonic energy to citrate did not further enhance phosphate release. This result strongly supports a ligand-promoted dissolution mechanism. Citrate acts as a powerful chelating agent, capable of sequestering metal ions (Ca^2+^ and Mg^2+^) [13]. We hypothesize that the removal of these metal ‘glues’ destabilizes the solid phase—potentially including metal-phosphate complexes or EPS-bound minerals—consequently facilitating the release of bound phosphate into the aqueous phase [28]. For a comprehensive overview of changes in other ionic species (e.g., Na^+^, K^+^, NH_4_^+^, Cl^−^, and SO_4_^2−^), please refer to Table A1. The fact that SC alone achieved similar efficacy to T/SC implies that this chemical substitution process is thermodynamically favorable and does not require additional physical energy to proceed.

### 3.2. Enhancement of VFA Production and Acidification Efficiency

**Figure 3:**
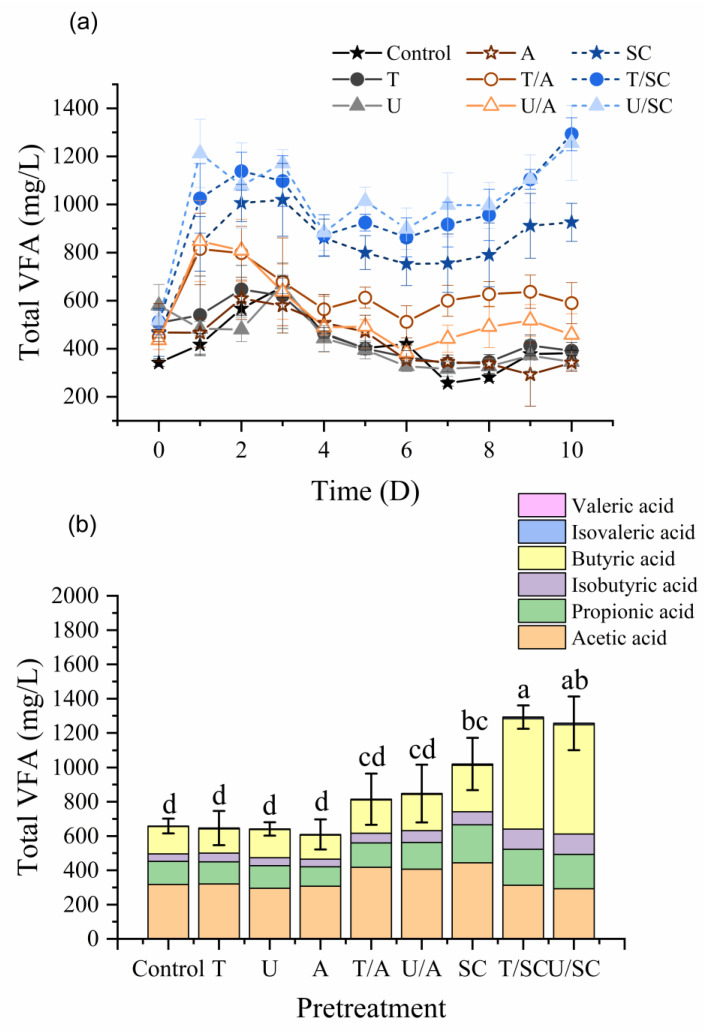
Effects of pretreatments on (**a**) total VFA production and (**b**) maximum yield and composition. Stacked bars show individual VFAs; bar height indicates total concentration. Mean ± SD (*n* = 6). Different letters denote significant differences (*p* < 0.05, Tukey’s HSD). T = thermal; U = ultrasonic; A = alkaline; SC = sodium citrate.

To elucidate the metabolic mechanisms driving acidification, the distribution of individual VFAs at their respective peak production times was analyzed (Figure 3a). The results revealed a distinct divergence in fermentation pathways, heavily influenced by the pretreatment strategy. For the Control, Physical (T, U), and Alkaline (A, T/A, U/A) groups, the fermentation process was characterized by a rapid but short-lived acidification phase. These groups achieved their maximum VFA concentrations between Day 1 and Day 3, with acetic acid being the predominant product. The dominance of acetate in these groups suggests a classic acetogenesis pathway in which solubilized substrates are rapidly converted to acetate [29,30]. However, the inability of these groups to sustain VFA accumulation implies that the fermentation was severely constrained, likely due to substrate depletion or the inhibition of specific microbial guilds by high pH in the alkaline-treated reactors.

In contrast, the T/SC and U/SC groups exhibited a unique dual-stage fermentation profile, corresponding to a massive enhancement in overall yield. Stage I (Days 1–3): An initial peak dominated by acetic acid was observed across all citrate-supplemented groups, accounting for approximately 47–48% of the total VFA. This aligns with the rapid biodegradation of citrate, which is metabolized to acetate and formate via oxaloacetate and pyruvate intermediates under anaerobic conditions [31].

**Figure 4:**
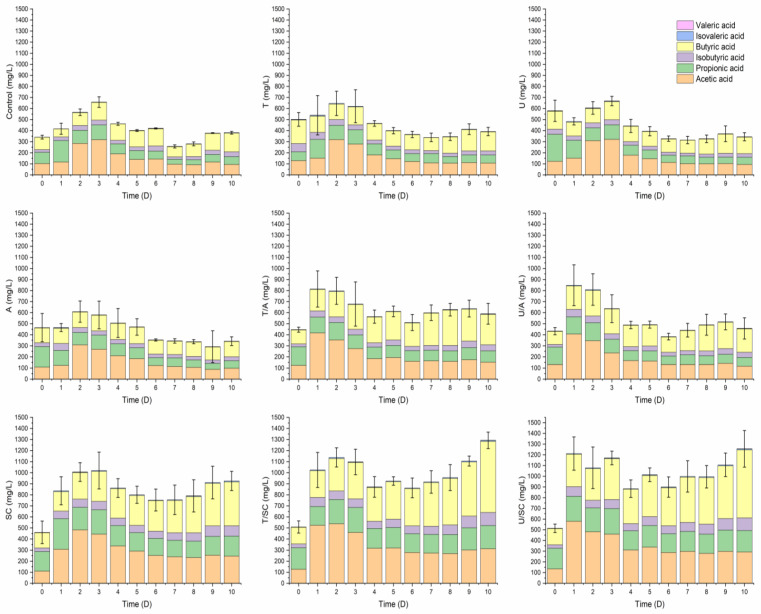
Temporal evolution of volatile fatty acid (VFA) composition across different pretreatment groups from Day 1 to Day 10, illustrating the natural metabolic shift towards butyrate dominance. Mean ± SD (*n* = 6). T = thermal; U = ultrasonic; A = alkaline; SC = sodium citrate.

Stage II (Day 10): A second, higher peak emerged on Day 10, wherein the VFA profile shifted towards a butyrate-dominant composition. The concentration of butyric acid surged significantly, accounting for roughly half of the total VFA. Based on the consistent temporal shift observed across treatment groups (Figure 4), this transition towards butyrate synthesis likely reflects the inherent natural succession of the sludge microbiome during extended fermentation [30]. The critical contribution of the T/SC strategy lies in its superior solubilization efficiency, which supplied an unprecedented abundance of bioavailable substrates. This massive carbon influx allowed the native acidogens to maximize their butyrate-forming potential without being prematurely constrained by substrate depletion. Furthermore, the elevated salinity from sodium citrate (~7000 mg/L of Na^+^, Table A1) likely provided a selective advantage by inhibiting methanogenic activity, effectively preventing VFA consumption [32]. The dominance of butyrate is highly desirable. Within the framework of a circular bioeconomy, butyrate serves as a premium precursor for manufacturing sustainable industrial products, such as eco-friendly bioplastics (e.g., polyhydroxyalkanoates, PHA), offering significantly greater economic value compared to acetate [33,34]. Furthermore, beyond bioplastics, this VFA-rich stream is highly versatile; it can be directly routed to downstream anaerobic digesters to significantly boost biogas (methane) production, or utilized as an easily biodegradable carbon source to enhance biological nutrient removal (denitrification) in the wastewater treatment plant.

### 3.3. Analysis of Main Effects and Interactions via Two-Way ANOVA

A two-way ANOVA was conducted to statistically distinguish the individual contributions of physical (P) and chemical (C) pretreatments, as well as their interactive effects (P × C), on sludge disintegration and acidification (Table 3). For organic solubilization indicators (*DD*_COD_, PN, and PS), both the main effects (P and C) and their interaction (P × C) were statistically significant (*p* < 0.05). Notably, the Chemical factor exhibited the highest F-value (*F* = 959), followed by the Physical factor (*F* = 252) and the Interaction term (*F* = 115), indicating that chemical disruption was the dominant driver. The significance of the interaction term supports a synergistic mechanism: the physical energy (thermal or ultrasonic) disrupts the floc structure, reducing particle size and increasing the specific surface area. This physical accessibility enhances the mass transfer of the chemical agents (citrate or alkali), allowing them to penetrate deeper into the matrix to chelate metals or saponify lipids [28]. Consequently, the combined efficiency exceeds the simple sum of the individual effects.

The analysis of ion release revealed a subtle but critical difference between calcium and magnesium. In calcium, although physical treatment alone was insignificant (*p* = 0.23), the interaction term was significant (*p* < 0.05, *F* = 4). This suggests that while heat/ultrasound cannot break Ca-bridges directly, it plays a crucial enabling role by exposing the calcium-binding sites to the chelating agents, thereby amplifying the chemical extraction efficiency. In contrast, the interaction term for Mg^2+^ was insignificant (*p* = 0.52), suggesting a purely chemical effect. This is supported by the overwhelmingly high F-value of the Chemical factor (*F* = 120) compared to the negligible *F*-value of the Physical factor (*F* = 3). This implies that magnesium release is almost exclusively governed by the chemical equilibrium (chelation strength) and is less dependent on physical structural loosening. This aligns with the observation in Figure 2, where citrate alone achieved Mg^2+^ release comparable to the combined T/SC treatment.

Regarding the final VFA yield, both physical and chemical factors were significant drivers (*p* < 0.05). However, the interaction term was marginally insignificant (*p* = 0.06). This statistical outcome points to an additive effect. It suggests that physical pretreatment (enhancing the hydrolysis rate) and citrate pretreatment (enhancing substrate quality and preventing P limitation) operate through relatively independent mechanisms. The total VFA enhancement in the T/SC group is essentially the summation of the kinetic benefits provided by thermal energy and the metabolic benefits provided by citrate chelation.

### 3.4. Preliminary Economic Feasibility and Practical Implications

To evaluate the practical viability of the T/SC pretreatment, a preliminary economic assessment was conducted. It should be noted that this assessment is highly conservative; it focuses solely on the direct operational expenditures (OPEX) versus the product revenue, deliberately excluding the cost savings from reduced sludge disposal volumes.

Based on standard thermodynamic calculations and current industrial electricity rates (0.14 USD/kWh), the thermal energy required to heat 1 metric ton of wet sludge to 121 °C costs approximately 18.5 USD. This mild thermal hydrolysis requires significantly less energy input compared to high-temperature pyrolysis (>400 °C), conventionally used for livestock sludge [35]. Combined with the bulk cost of sodium citrate (~11.5 USD), the total OPEX increase is highly controllable. Although T/SC requires an initial chemical and thermal investment, this is fundamentally offset by multiple streams of economic return. Directly, the pretreatment not only doubled the total VFA yield but also facilitated the sustained accumulation of a butyrate-dominant mixture. Given that butyrate possesses a significantly higher market value (typically >2000 USD/ton) as a green chemical building block compared to acetate (~600 USD/ton) [34], the direct economic return is highly promising. Indirectly, the profound disintegration of the EPS matrix and the unzipping of the floc structure substantially improve sludge dewaterability and reduce the final volume of residual sludge. In full-scale operations, this translates to massive cost savings in sludge transport and disposal, which often account for up to 50% of the total operating costs in wastewater treatment plants. Additionally, if the facility opts to utilize the VFA-rich liquid for methanogenesis rather than chemical extraction, the enhanced biogas yield can be converted into electricity and heat via a combined heat and power (CHP) system, thereby partially or fully offsetting the thermal energy required for the initial 121 °C hydrolysis. Overall, this T/SC approach provides a scientifically sound and economically viable pathway to optimize the manure management chain.

Furthermore, an additional operational advantage of the T/SC process is the substantial reduction in sludge viscosity following the unzipping of the EPS network and floc disintegration. In full-scale operations, this rheological improvement directly translates to significant energy savings in pumping and mechanical mixing, further offsetting the initial chemical investment.

## 4. Conclusions

This study demonstrated that coupling thermal hydrolysis with citrate chelation (T/SC) is a superior strategy for valorizing metal-rich swine wastewater sludge. T/SC pretreatment achieved the highest disintegration degree (12.37%) and biopolymer solubilization, significantly outperforming traditional alkaline–thermal methods. The macroscopic data suggest that the superior performance of citrate is highly associated with its capability to sequester both Ca^2+^ and Mg^2+^, which is hypothesized to disrupt the ionic bridges stabilizing the floc structure. In contrast, the poor performance of alkaline treatment implies a limitation likely caused by the rapid reprecipitation of magnesium and phosphate. Consequently, T/SC not only enhanced total VFA production 2-fold (1293 mg/L) but also induced a favorable metabolic shift from acetogenesis to chain elongation, yielding a butyrate-rich product. Furthermore, preliminary economic assessment indicates that the significant market value of the generated butyrate fundamentally offsets the operational expenditures of sodium citrate and thermal heating. Overall, these findings strongly suggest that targeting and overcoming ionic bridging serves as a critical pathway for maximizing efficient resource recovery from specific metal-laden organic wastes like swine sludge.

## Figures and Tables

**Table 1:** Characteristics of livestock sludge (*n* = 6).

Characteristics	Livestock Sludge
pH	6.86 ± 0.18
EC (mS/cm)	0.847 ± 0.093
TS (g/L)	97 ± 2
VS (g/L)	63 ± 1
SOCD (mg/L)	580 ± 44
TCOD (mg/L)	53,591 ± 1333
PN (mg/L)	14.4 ± 2.7
PS (mg/L)	4.1 ± 0.8

Note: EC = Electrical Conductivity. TS = total solid; VS = Volatile solid; SCOD = Soluble Chemical Oxygen Demand; TCOD = Total Chemical Oxygen Demand; PN = Soluble Protein; PS = Soluble Polysaccharide. Mean ± SD.

**Table 2:** Conditions of livestock sludge pretreatments.

Pretreatment	Conditions	Treating Time
Thermal (T)	121 °C	30 min
Ultrasonic (U)	40 kHz, 300 W	60 min
Alkaline (A)	pH = 12	60 min
Sodium citrate (SC)	0.3 g/g-TS	60 min
Thermal and Sodium citrate (T/SC)	0.3 g/g-TS, 121 °C	30 min, 60 min
Ultrasonic and Sodium citrate (U/SC)	0.3 g/g-TS, 40 kHz, 300 W	60 min
Thermal and Alkaline (T/A)	pH = 12, 121 °C	30 min, 60 min
Ultrasonic and Alkaline (U/A)	pH = 12, 40 kHz, 300 W	60 min

**Table 3:** Two-way ANOVA *p*-values and F-value for solubilization indicators and VFA production, evaluating the effects of physical pretreatment (P), chemical pretreatment (C), and their interaction (P × C).

Indicator		Physical (P)	Chemical (C)	Interaction (P × C)	Interpretation of Joint Effect
Solubilization					
*DD*_COD_	*p*-value	<0.05	<0.05	<0.05	Synergistic
	*F*-value	252	959	115	
PN and PS	*p*-value	<0.05	<0.05	<0.05	Synergistic
	*F*-value	259	531	116	
Ion Release					
Ca^2+^	*p*-value	0.23	<0.05	<0.05	Synergistic
	*F*-value	1.5	716	4	
Mg^2+^	*p*-value	0.08	<0.05	0.52	Additive
	*F*-value	3	120	1	
PO_4_^3−^	*p*-value	<0.05	<0.05	<0.05	Synergistic
	*F*-value	12	372	4	
Product					
Max VFAs	*p*-value	<0.05	<0.05	0.06	Additive
	*F*-value	9	87	2	

Note: *DD*_COD_ = Disintegration Degree, PN = Soluble Protein, PS = Soluble Polysaccharide, VFAs = Volatile Fatty Acids.

## Data Availability

The original contributions presented in this study are included in the article. Further inquiries can be directed to the corresponding author.

## Appendix A

**Table A1:** Changes in cation and anion composition in sludge with various pretreatments.

**Pretreatment**	**Cations (mg/L)**				
	**Na^+^**	**NH_4_^+^**	**K^+^**	**Ca^2+^**	**Mg^2+^**
Control	51.8 ± 7.7	90.4 ± 32.4	67.8 ± 3.5	58.0 ± 16.0	19.0 ± 7.0
T	91.1 ± 61.8	110.3 ± 23.8	71.7 ± 18.2	65.8 ± 29.0	15.6 ± 5.5
U	60.9 ± 16.8	100.2 ± 24.3	61.9 ± 12.7	139.1 ± 101.2	15.7 ± 3.7
A	1577.3 ± 369.3	150.3 ± 28.8	86.8 ± 17.9	642.2 ± 73.0	73.2 ± 23.9
T/A	1261.8 ± 41.2	112.9 ± 12.4	72.2 ± 6.2	681.8 ± 117.4	50.8 ± 10.6
U/A	1130.7 ± 160.3	103.5 ± 20.5	65.8 ± 9.7	517.7 ± 90.7	47.1 ± 10.2
SC	7498.0 ± 1556.9	141.0 ± 24.4	88.2 ± 23.1	1077.3 ± 48.8	116.5 ± 27.5
T/SC	6876.4 ± 1648.6	138.3 ± 20.5	86.7 ± 13.6	1085.6 ± 58.7	114.0 ± 21.4
U/SC	7198.4 ± 1571.0	128.4 ± 22.7	78.2 ± 22.8	1041.9 ± 30.4	104.1 ± 21.4
**Pretreatment**	**Anions (mg/L)**				
	**Cl^−^**	**NO_2_^−^**	**NO_3_^−^**	**PO_4_^3−^**	**SO_4_^2−^**
Control	49.8 ± 4.2	ND	ND	73.6 ± 3.2	13.0 ± 4.0
T	52.8 ± 7.1	ND	ND	79.2 ± 15.9	25.7 ± 10.9
U	45.9 ± 6.5	ND	ND	68.8 ± 21.5	28.3 ± 7.3
A	2359.9 ± 131.7	ND	ND	125.8 ± 27.4	9.2 ± 3.9
T/A	2845.0 ± 550.8	ND	ND	295.7 ± 39.9	14.0 ± 5.0
U/A	2492.7 ± 573.4	ND	ND	200.6 ± 40.6	21.0 ± 12.3
SC	75.1 ± 22.0	ND	ND	489.0 ± 52.2	33.3 ± 9.0
T/SC	41.3 ± 11.4	ND	ND	569.7 ± 88.1	23.2 ± 1.4
U/SC	38.8 ± 5.1	ND	ND	555.7 ± 74.2	17.9 ± 13.0

Note: T = thermal; U = ultrasonic; A = alkaline; SC = sodium citrate; and ND = not detected (<0.1 mg/L). Values represent mean ± SD.

## References

[1] Fang W., Zhang X., Zhang P., Wan J., Guo H., Ghasimi D.S.M., Morera X.C., Zhang T. (2020). Overview of key operation factors and strategies for improving fermentative volatile fatty acid production and product regulation from sewage sludge. J. Environ. Sci..

[2] Ramos-Suarez M., Zhang Y., Outram V. (2021). Current perspectives on acidogenic fermentation to produce volatile fatty acids from waste. Rev. Environ. Sci. Bio/Technol..

[3] Appels L., Baeyens J., Degrève J., Dewil R. (2008). Principles and potential of the anaerobic digestion of waste-activated sludge. Prog. Energy Combust. Sci..

[4] Richard E.N., Hilonga A., Machunda R.L., Njau K.N. (2019). A review on strategies to optimize metabolic stages of anaerobic digestion of municipal solid wastes towards enhanced resources recovery. Sustain. Environ. Res..

[5] Carrère H., Dumas C., Battimelli A., Batstone D.J., Delgenès J.P., Steyer J.P. (2010). Pretreatment methods to improve sludge anaerobic degradability: A review. J. Hazard. Mater..

[6] Zhen G., Lu X., Kato H., Zhao Y., Li Y.Y. (2017). Overview of pretreatment strategies for enhancing sewage sludge disintegration and subsequent anaerobic digestion: Current advances, full-scale application and future perspectives. Renew. Sustain. Energy Rev..

[7] Yen K.W., Chen W.C., Su J.-J. (2024). Recovery of copper and zinc from livestock bio-sludge with an environmentally friendly organic acid extraction. Animals.

[8] Higgins M.J., Novak J.T. (1997). The effect of cations on the settling and dewatering of activated sludges: Laboratory results. Water Environ. Res..

[9] Sobeck D.C., Higgins M.J. (2002). Examination of three theories for mechanisms of cation-induced bioflocculation. Water Res..

[10] Chen Y., Jiang S., Yuan H., Zhou Q., Gu G. (2007). Hydrolysis and acidification of waste activated sludge at different pHs. Water Res..

[11] Bi W., Li Y., Hu Y. (2014). Recovery of phosphorus and nitrogen from alkaline hydrolysis supernatant of excess sludge by magnesium ammonium phosphate crystallization. Bioresour. Technol..

[12] Ye Y., Ngo H.H., Guo W., Liu Y., Li J., Liu Y., Zhang X., Jia H. (2017). Insight into chemical phosphate recovery from municipal wastewater. Sci. Total Environ..

[13] Yang G., Wang J. (2019). Enhancing biohydrogen production from waste activated sludge disintegrated by sodium citrate. Fuel.

[14] Fang W., Yang Y., Wang C., Zhang P. (2022). Enhanced volatile fatty acid production from anaerobic fermentation of waste activated sludge by combined sodium citrate and heat pretreatment. J. Environ. Chem. Eng..

[15] Liu X.L., Liu H., Du G.C., Chen J. (2009). Improved bioconversion of volatile fatty acids from waste activated sludge by pretreatment. Water Environ. Res..

[16] Xiao B., Liu C., Liu J., Guo X. (2015). Evaluation of the microbial cell structure damages in alkaline pretreatment of waste activated sludge. Bioresour. Technol..

[17] Tampio E.A., Blasco L., Vainio M.M., Kahala M.M., Rasi S.E. (2019). Volatile fatty acids (VFAs) and methane from food waste and cow slurry: Comparison of biogas and VFA fermentation processes. GCB Bioenergy.

[18] Rice E.W., Baird R.B., Eaton A.D., Clesceri L.S. (2012). Standard Methods for the Examination of Water and Wastewater.

[19] Kim D.H., Jeong E., Oh S.E., Shin H.S. (2010). Combined (alkaline+ultrasonic) pretreatment effect on sewage sludge disintegration. Water Res..

[20] Frølund B., Griebe T., Nielsen P.H. (1995). Enzymatic activity in the activated-sludge floc matrix. Appl. Microbiol. Biotechnol..

[21] DuBois M., Gilles K.A., Hamilton J.K., Rebers P.T., Smith F. (1956). Colorimetric method for determination of sugars and related substances. Anal. Chem..

[22] Tchobanoglous G., Stensel H.D., Tsuchihashi R., Burton F.L. (2014). Wastewater Engineering: Treatment and Resource Recovery.

[23] Wee C.Y., Su J.-J. (2019). Biofuel produced from solid-state anaerobic digestion of dairy cattle manure in coordination with black soldier fly larvae decomposition. Energies.

[24] Mata-Alvarez J., Macé S., Llabrés P. (2000). Anaerobic digestion of organic solid wastes. An overview of research achievements and perspectives. Bioresour. Technol..

[25] Yan Y., Feng L., Zhang C., Wisniewski C., Zhou Q. (2010). Ultrasonic enhancement of waste activated sludge hydrolysis and volatile fatty acids accumulation at pH 10.0. Water Res..

[26] Tyagi V.K., Lo S.L., Appels L., Dewil R. (2014). Ultrasonic treatment of waste sludge: A review on mechanisms and applications. Crit. Rev. Environ. Sci. Technol..

[27] Wang X., Zhang M., Zhou Z., Qu T., Ran J., Zhang J., Li X., Zhang L., Zhang A. (2024). Effect of extracellular polymeric substances removal and re-addition on anaerobic digestion of waste activated sludge. J. Water Process Eng..

[28] Gu S., Wang N., Huang Y., Xiang Z., Yang Q. (2025). Experimental study on the elution of ultrasonically coupled sodium citrate–enhanced oily sludge. Chem. Eng. Res. Des..

[29] McCarty P.L. (1964). Anaerobic waste treatment fundamentals. Public Works.

[30] Zhou M., Yan B., Wong J.W.C., Zhang Y. (2018). Enhanced volatile fatty acids production from anaerobic fermentation of food waste: A mini-review focusing on acidogenic metabolic pathways. Bioresour. Technol..

[31] Bott M. (1997). Anaerobic citrate metabolism and its regulation in enterobacteria. Arch. Microbiol..

[32] Chen Y., Cheng J.J., Creamer K.S. (2008). Inhibition of anaerobic digestion process: A review. Bioresour. Technol..

[33] Agler M.T., Wrenn B.A., Zinder S.H., Angenent L.T. (2011). Waste to bioproduct conversion with undefined mixed cultures: The carboxylate platform. Trends Biotechnol..

[34] Bhatia S.K., Yang Y.-H. (2017). Microbial production of volatile fatty acids: Current status and future perspectives. Rev. Environ. Sci. Bio/Technol..

[35] Lee S., Kim Y.M., Siddiqui M.Z., Park Y.K. (2021). Different pyrolysis kinetics and product distribution of municipal and livestock manure sewage sludge. Environ. Pollut..

